# Meta-analysis with a single study

**DOI:** 10.1177/09622802251380628

**Published:** 2025-10-09

**Authors:** Erik van Zwet, Witold Wiȩcek, Andrew Gelman

**Affiliations:** 1 Department of Biomedical Data Sciences, 4501 Leiden University Medical Center, Leiden, the Netherlands; 2 Development Innovation Lab, 2462 University of Chicago, Chicago, IL, USA; 3 Department of Statistics and Department of Political Science, 5798 Columbia University, New York, NY, USA

**Keywords:** Meta-analysis, heterogeneity, Bayes, empirical Bayes

## Abstract

Effect sizes typically vary among studies of the same intervention. In a random effects meta-analysis, this source of variation is taken into account, at least to some extent. However, when we have only one study, the heterogeneity remains hidden and unaccounted for. Treating the study-level effect as if it is the population-level effect leads to underestimation of the uncertainty. We propose an empirical Bayesian approach to address this problem. We start by estimating the distribution of the population-level effects and heterogeneity among 1635 meta-analyses from the Cochrane Database of Systematic Reviews. Using both synthetic data and cross-validation, we assess the consequences of using these estimated distributions as prior information for the analysis of single trials. We find that our Bayesian “meta-analyses of single studies” perform much better than naively assuming non-varying effects. The prior on the heterogeneity results in better quantification of the uncertainty. The prior on the treatment effect substantially reduces the mean squared error both for estimating the study-level and population-level effects. For the latter, this reduction is equivalent to doubling the sample size.

## 1. Introduction

It is common practice to interpret the observed treatment effect of a clinical trial as an estimate of an underlying true treatment effect. This is reasonable. We claim that it is also common to tacitly, perhaps even unconsciously, assume that this true effect is an immutable property of the treatment. Thus, one might speak of “the” effect of a particular treatment or intervention, and expect the same effect in another study about the same treatment. However, there are many good reasons to expect variation among the underlying effects from differences between study populations, application of the treatment and measurement protocols among other factors. There is also much empirical evidence of effect heterogeneity from random effects meta-analyses.^1–3^ Failure to take this into account when interpreting the results of a trial will lead to underestimation of the uncertainty about the treatment effect.

Recall that meta-analysis is a quantitative approach for combining inferences from multiple studies. Besides providing an estimate of the average or “population-level” effect of the treatment, together with some measure of uncertainty such as a standard error or confidence interval, a meta-analysis also provides an estimate of the variation of underlying effects of the individual studies. Understanding and quantifying heterogeneity is an important aspect of meta-analysis, as it can influence the interpretation of the overall results.

The Cochrane Database of Systematic Reviews (CDSR) is a globally respected collection of evidence-based healthcare information, comprising rigorous and comprehensive systematic reviews on diverse medical topics, with many of those reviews including meta-analyses.^4^ The database is continuously updated, offering the latest evidence to inform clinical practice, policy decisions, and research priorities. It adheres to strict quality standards, undergoes peer review, and includes open-access summaries for wider accessibility.

We will assume the standard random effects meta-analysis model. That is, we assume the following two-part hierarchical (or multilevel) model for the j -th study in a collection of studies of the same treatment

(1) $$\begin{array}{rl} \beta_{j} & =\mu +u_{j} \end{array}$$

(2) $$\begin{array}{rl} b_{j} & =\beta_{j}+\varepsilon_{j} \end{array}$$

The error terms $u_{j}∼\text{normal}(0,\tau )$ and $\varepsilon_{j}∼\text{normal}(0,s_{j})$ are assumed to be independent. The first part (1) models the variation among studies of the same treatment. Here, μ is the population-level average treatment effect in the (hypothetical) superpopulation of similar studies and $\beta_{j}$ is the study-level effect in the j -th individual study. The variance $\tau^{2}$ is referred to as the heterogeneity. The second part (2) models the uncertainty of the estimates from each individual study. We assume that the estimate $b_{j}$ from the j -th study is unbiased and normally distributed with standard error $s_{j}$ .

When we have only one study, we cannot separate the two error terms. Without additional assumptions, there is nothing more to do than estimate $\beta_{1}$ by $b_{1}$ with confidence interval $b_{1}\pm 1.96\;s_{1}$ . If we make the extra assumption that there is no heterogeneity, i.e. τ=0 , then we can also estimate the population average effect μ by $b_{1}$ with confidence interval $b_{1}\pm 1.96\;s_{1}$ . As we discussed, we believe that this extra assumption is commonly—if implicitly—made when interpreting the result of a single trial. However, τ is typically not zero, so this is not correct.

Instead of assuming that τ is zero, we propose a Bayesian approach with the prior information based on a hierarchical model fit to the CDSR. First, we estimate the distributions of both μ and τ across the CDSR and use those as empirical priors. Estimating one or both of these distributions from collections of meta-analysis is not new, see for instance.^2,3,5^ We then proceed to use the R package baggr^6^ to use these distributions as prior information for Bayesian inference. We call this meta-analysis with a single trial. The baggr package is based on Stan, a state-of-the-art platform for statistical modeling and high-performance statistical computation.^7^

We compare the performance across the trials of the CDSR of our Bayesian approach to the naive approach of assuming that τ is zero. To do this, we construct a “synthetic copy” of the CDSR and also use a kind of leave-one-out cross-validation.

## 2. Meta-analysis with a single trial

### 2.1. Estimating the distributions of μ and τ

Many of the systematic reviews in the CDSR are accompanied by meta-analyses. These data have been processed and made available by Schwab (2020).^8^ Since the trials in the same meta-analysis are evaluating the same intervention, we can use the data from the CDSR to study how the effects of the same treatment vary across multiple studies.

We start by selecting trials with either a binary or numerical primary efficacy outcome; these comprise 97% of the trials in the CDSR. To make the effect sizes of the binary and numerical outcomes comparable, we quantify the treatment effect on the probit scale for all binary outcomes, and as the standardized mean difference (SMD) for all continuous outcomes. Next, we select all meta-analyses with at least 5 individual studies. Meta-analyses with fewer studies have little information about heterogeneity and discarding them reduces computation time. This leaves 18,368 unique trials from 1635 meta-analyses. We consider the following hierarchical model. For the j -th individual study in the i -th meta-analysis, we assume

(3) $$\begin{array}{rl} \beta_{ij} & =\mu_{i}+u_{ij} \end{array}$$

(4) $$\begin{array}{rl} b_{ij} & =\beta_{ij}+\varepsilon_{ij} \end{array}$$

where $u_{ij}∼\text{normal}(0,\tau_{i})$ and $\varepsilon_{ij}∼\text{normal}(0,s_{ij})$ . All the $u_{ij}$ and $\varepsilon_{ij}$ are assumed to be independent. From the CDSR, we obtain the pairs $\left(b_{ij},s_{ij}\right)$ . We define the z -values for each pair as $z_{ij}=b_{ij}/s_{ij}$ .

We want to estimate the distribution of the $\mu_{i}$ and $\tau_{i}$ across the CDSR. We assume that the $\mu_{i}$ follow a generalized t distribution and that the $\tau_{i}$ are lognormally distributed. Moreover, we assume that the $\mu_{i}$ and $\tau_{i}$ are independent.

To estimate the five parameters of our model (the mean, scale and degrees of freedom of the generalized t -distribution and the mean and standard deviation of the normal distribution) we use a Bayesian approach with uniform priors on the two means, the degrees of freedom, and the logarithm of the two scale parameters. The posterior distributions of the parameters are approximately normal, so that the posterior means are approximately equal to the maximum likelihood estimates. We show these estimates in the top row of Table 1.

**Table 1.** Estimated parameters of the t distribution of μ and the normal distribution of logτ . In the bottom row, we restrict the mean of μ to be zero. These estimates are based on 18,368 unique trials from 1635 meta-analyses.

	t distribution of μ			Normal distribution of logτ	
	Center	Scale	*df*	Mean	Std Dev
Unrestricted	−0.11	0.37	5.30	−1.82	0.89
Zero mean	0.00	0.37	5.18	−1.82	0.90

We can provide some context for these estimated distributions by recalling the tentative classification of effect sizes by Cohen according to which SMD values of 0.2–0.5 are considered small, 0.5–0.8 are considered medium, and >0.8 are considered large.^9,10^ The estimated t -distribution of μ implies that the median of the absolute value of μ is 0.28 (IQR: 0.13–0.50). In other words, 75% of the population-level average effects may be considered to be small. The median of τ is 0.16 (IQR: 0.09–0.30). So, we find that the heterogeneity is roughly on the order of half the effect size.

We also fit the model restricting the center of the distribution of effect sizes $\mu_{i}$ to be 0, and show the resulting estimates in the bottom row of Table 1. We will use this distribution as our prior, because it ensures that we treat positive and negative effect estimates equally. This seems fair because, to some extent, the sign of the effect estimate is arbitrary. For example, one could either consider the proportion of patients alive or dead after one year, but this choice should not have any material effect on our inferences about the effectiveness of the treatment.

### 2.2. An example

We demonstrate our approach with a small example. Suppose that we have a single trial with a numerical outcome, with estimated SMD of b=0.7 . Suppose that the standard error also 0.7, so that the z -value is 1 and the p -value is 0.32. The 95% confidence interval is (−0.7,2.1) .

We can use the R package baggr to incorporate the prior information that is represented in Table 1. We first use a flat prior for the population-level effect μ and an informative prior for the heterogeneity.


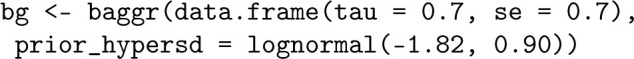


We find that the posterior distribution of the study-level effect (the true effect in the trial) β remains numerically the same. That is, the posterior mean is $\hat{\beta }=0.7$ with 95% posterior interval from − 0.7 to 2.1. Now we also obtain an estimate of the population-level effect (the average effect in similar trials) μ . The posterior mean is $\hat{\mu }=0.7$ with 95% posterior interval (−0.9,2.2) . The prior information about the heterogeneity is reflected in a wider interval.

Next, we also incorporate information about the population-level effect μ . We use the following call to baggr.


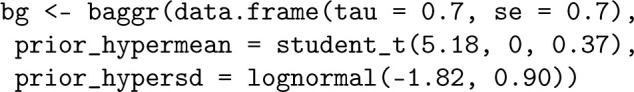


The zero-mean prior for μ induces considerable shrinkage. We find that the posterior mean of β is now $\hat{\beta }=0.24$ with 95% posterior interval from −0.5 to 1.1. The posterior mean of μ becomes $\hat{\mu }=0.17$ with 95% posterior interval (−0.5,1.0) . Moreover, using the code from the Supplemental Appendix, we find that the posterior probability that β is positive is 0.72, while the posterior probability that μ is positive is 0.67.

### 2.3. The probability of the correct sign

**Figure 1.**
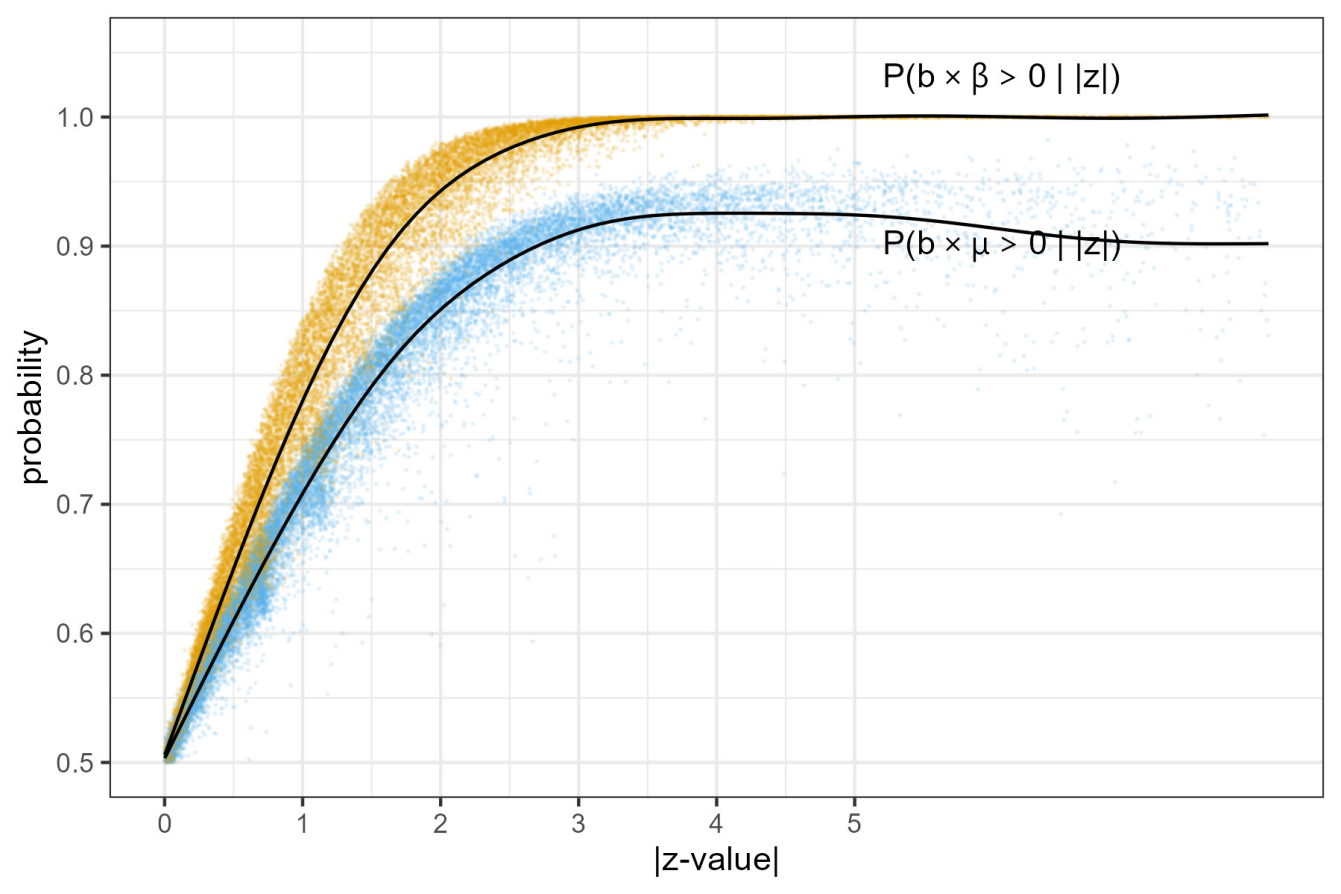
Conditional probabilities of b having the same sign as β or μ given the observed absolute z -value.

Next, we use baggr to perform all 18,368 meta-analyses with one trial and compute the posterior probabilities that the observed $b_{ij}$ has the same sign as $\beta_{ij}$ and $\mu_{i}$ . We plot the observed absolute z -values $|z_{ij}|=|b_{ij}/s_{ij}|$ versus these probabilities in Figure 1. We fitted smooth regression curves which can be interpreted as conditional probabilities given the observed absolute z -value. We see that a single trial never provides certainty about the sign of μ .

## 3. Performance of the method

### 3.1. Building a synthetic CDSR

We construct a “synthetic” CDSR to evaluate the performance of our Bayesian approach and compare it to the naive approach of assuming that τ is 0. To generate the synthetic database we perform the following steps:

1. Sample $\mu_{i}^{*}$ and $\tau_{i}^{*}$ ( i=1,2,…,1635 ) from the estimated distribution in the top row of Table 1. We are not restricting the $\mu^{*}$ to be 0 on average.
2. To induce dependence between the pairs $\mu_{i}^{*}$ and $\tau_{i}^{*}$ , sort them in the same order as the maximum likelihood estimates of $\mu_{i}$ and $\tau_{i}$ , which we obtain by performing the meta-analyses of the CDSR data. Break any ties at random.
3. Sample independent $\beta_{ij}^{*}$ ( i=1,2,…,1635 and $j=1,2,\ldots ,n_{i}$ ) from the normal distribution with mean $\mu_{i}^{*}$ and standard deviation $\tau_{i}^{*}$ .
4. Sample $b_{ij}^{*}$ from the normal distribution with mean $\beta_{ij}^{*}$ and the *observed* standard deviation $s_{ij}$ . Set $z_{ij}^{*}=b_{ij}^{*}/s_{ij}$ .

**Figure 2.**
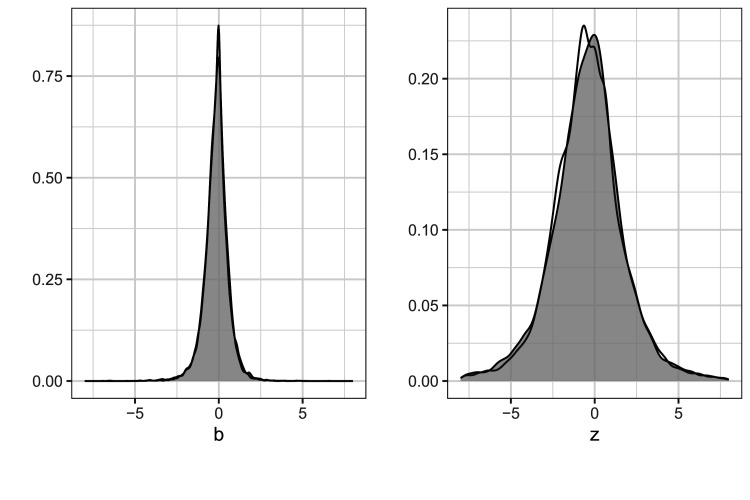
Observed and simulated distributions of 18,368 estimates and z -values.

For i=1,2,…,1635 and $j=1,2,\ldots ,n_{i}$ , we now have simulated sets $\left(\mu_{i}^{*},\tau_{i}^{*}\right)$ and $\left(\beta_{ij}^{*},b_{ij}^{*},s_{ij}\right)$ that should be similar to the original CDSR. We can confirm this to some extent by comparing the distribution of the observed $b_{ij}$ and $z_{ij}$ to the simulated $b_{ij}^{*}$ and $z_{ij}^{*}$ in Figure 2.

### 3.2. Estimating the effect in the trial

Once again we use baggr to perform all meta-analyses with one trial in the simulated dataset to estimate $\beta_{ij}^{*}$ and $\mu_{ij}^{*}$ by their respective posterior means ${\hat{\beta }}_{ij}^{*}$ and ${\hat{\mu }}_{ij}^{*}$ . We also construct uncertainty (“credible”) limits. We then compare to the naive approach where we estimate both quantities by $b_{ij}^{*}$ with interval $\left(b_{ij}^{*}\pm 1.96\times s_{ij}\right)$ .

In Table 2, we show the root mean squared error (RMSE), bias of the magnitude, and coverage of these two estimators. To be precise, for the naive approach we compute

(5) $$\begin{array}{rl} \text{mean squared error} & =\frac{1}{18,368}\sum_{i,j}(b_{ij}^{*}-\beta_{ij}^{*}{)}^{2} \end{array}$$

(6) $$\begin{array}{rl} \text{bias of the magnitude} & =\frac{1}{18,368}\sum_{i,j}|b_{ij}^{*}|-|\beta_{ij}^{*}| \end{array}$$

(7) $$\begin{array}{rl} \text{coverage} & =\frac{1}{18,368}\sum_{i,j}1_{|b_{ij}^{*}-\beta_{ij}^{*}|<1.96s_{ij}} \end{array}$$

**Table 2.** Performance of the unbiased estimators $b_{ij}^{*}$ and Bayesian estimators ${\hat{\beta }}_{ij}^{*}$ of the effects in the trial $\beta_{ij}$ . The right side of the table shows the average over the statistically significant trials, that is, $|z_{ij}^{*}|>1.96$ .

	Unconditional			Statistically significant		
Method	RMSE	Bias	Coverage	RMSE	Bias	Coverage
Unbiased	0.38	0.10	0.95	0.42	0.21	0.90
Bayes	0.29	−0.06	0.95	0.29	−0.01	0.95

RMSE: root mean squared error.

We compute the analogous quantities for the Bayesian approach. The left side Table 2 displays the three performance measures for all trials and the right side by averaging only over the statistically significant trials with $|z_{ij}^{*}|>1.96$ .

Over all trials, the bias of the magnitude for the naive estimator is 0.1, which is due to Jensen’s inequality; recall that the absolute value is a convex function. As expected, the coverage of the usual confidence interval equals its nominal level. The right side of the table shows that selection on significance increases the upward bias of the magnitude to 0.21. This is sometimes called the “winner’s curse.” This bias also causes the mean squared error to increase. Moreover, the usual confidence interval no longer reaches nominal coverage.

When we turn to our Bayesian approach, we find that the RMSE is substantially reduced compared to the unbiased estimator: the MSE is reduced from $0.38^{2}=0.14$ to $0.29^{2}=0.08$ , which would be equivalent to an increase of the sample size of more than 71%! Conditionally on statistical significance, the reduction of the RMSE is even greater.

The reductions in the RMSE are due to the “shrinkage” that is induced by the zero-mean prior for μ which implies a zero-mean prior for β . This pulls the unbiased naive estimate towards zero, that is $|{\hat{\beta }}_{ij}^{*}|<|b_{ij}^{*}|$ . We also see the effect of shrinkage in the reduction of the bias of the magnitude. On average across all the trials, the bias of the magnitude of the Bayesian estimator is −0.06 . When we condition on statistical significance, however, this bias is almost exactly offset by the winner’s curse. The coverage of the Bayesian uncertainty interval is nominal, both unconditionally and conditionally. The superior performance of a similar Bayesian estimator was seen by van Zwet et al.^11^

### 3.3. Estimating the population average effect

We also have two estimators for the population average effect $\mu_{i}^{*}$ , namely the naive estimator $b_{ij}^{*}$ and the Bayesian estimator ${\hat{\mu }}_{ij}^{*}$ . Again, we compute the RMSE, bias of the magnitude, and coverage both conditionally and unconditionally on statistical significance. We show the results in Table 3.

**Table 3.** Performance of the unbiased estimators $b_{ij}^{*}$ and Bayesian estimators ${\hat{\mu }}_{ij}^{*}$ of the population average effect $\mu_{i}^{*}$ . The right side of the table shows the average over the statistically significant trials, that is, $|z_{ij}^{*}|>1.96$ .

	Unconditional			Statistically significant		
Method	RMSE	Bias	Coverage	RMSE	Bias	Coverage
Unbiased	0.53	0.15	0.82	0.72	0.41	0.63
Bayes	0.35	−0.11	0.93	0.38	−0.03	0.94

RMSE: root mean squared error.

Essentially, the same observations apply to the results Table 3 as to those in Table 2. The reduction in the RMSE of the Bayesian estimator compared to the unbiased estimator is even more extreme. The MSE is reduced by more than a factor of 2 from $0.53^{2}=0.28$ to $0.35^{2}=0.12$ which is equivalent to more than doubling the sample size. Also, the bias of $|b_{ij}^{*}|$ as an estimator of $|\mu_{ij}^{*}|$ is large, especially for statistically significant trials. This bias is completely absent for the Bayesian estimator.

The coverage of the usual confidence interval is far below 95% both with and without conditioning on statistical significance. This is a direct result of not taking the heterogeneity into account. The coverage of the Bayesian uncertainty interval is close to nominal in both cases.

### 3.4. Graphical comparison

Tables 2 and 3 provide a broad overview of the performance of the naive and Bayesian estimators. We will now study the performance in some more detail both from the frequentist and Bayesian points of view. The frequentist point of view means that we condition on the true effects $\beta_{ij}^{*}$ and $\mu_{i}^{*}$ . The Bayesian point of view, on the other hand, means that we condition on the observed effect $b_{ij}^{*}$ .

We first consider the bias. The naive estimator $b_{ij}^{*}$ is unbiased both for $\beta_{ij}^{*}$ and $\mu_{i}^{*}$ so we do not need to study this further. We focus on the bias of the Bayesian estimators ${\hat{\beta }}_{ij}^{*}$ and ${\hat{\mu }}_{ij}^{*}$ which are both shrinkage estimators, in the sense that $|{\hat{\mu }}_{ij}^{*}|<|{\hat{\beta }}_{ij}^{*}|<|b_{ij}^{*}|$ .

**Figure 3.**
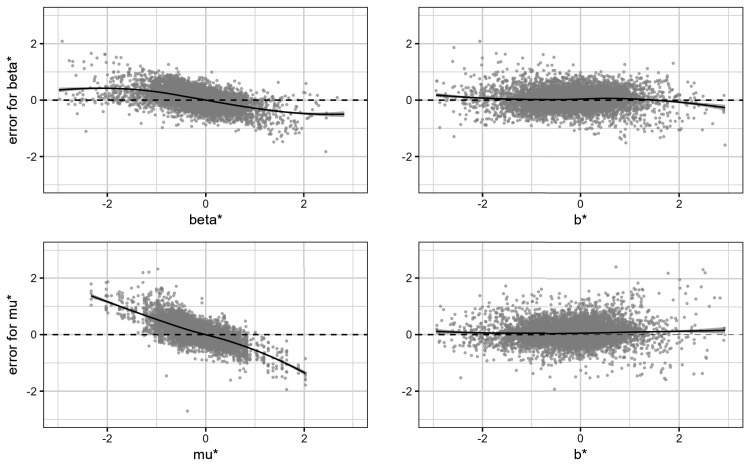
Bias of the proposed Bayesian estimators. The two top panels show ${\hat{\beta }}_{ij}^{*}-\beta_{ij}^{*}$ . The two bottom panels show ${\hat{\mu }}_{ij}^{*}-\mu_{i}^{*}$ . The two panels on the left side show the frequentist bias, while the two panels on the right side show the Bayesian bias.

In the top left panel of Figure 3, we plot the estimation errors ${\hat{\beta }}_{ij}^{*}-\beta_{ij}^{*}$ versus the true effects in the trials $\beta_{ij}^{*}$ . In the bottom left panel, we plot the errors ${\hat{\mu }}_{ij}^{*}-\mu_{i}^{*}$ versus the true pooled effects $\mu_{ij}^{*}$ versus. In the two right panels, we plot the same estimation errors, but in both cases we put the observed effects $b_{ij}^{*}$ on the x -axis. We added the loess regression curves to each of the four plots, which estimate the following conditional expectations:

$$\begin{array}{rl} & E({\hat{\beta }}_{ij}^{*}-\beta_{ij}^{*}|\beta_{ij}^{*})\;\text{(top left panel of Figure 3)}\\ & E({\hat{\mu }}_{ij}^{*}-\mu_{i}^{*}|\mu_{i}^{*})\;\text{(bottom left panel of Figure 3)}\\ & E({\hat{\beta }}_{ij}^{*}-\beta_{ij}^{*}|b_{ij}^{*})\;\text{(top right panel of Figure 3)}\\ & E({\hat{\mu }}_{ij}^{*}-\mu_{i}^{*}|b_{ij}^{*})\;\text{(bottom right panel of Figure 3)} \end{array}$$

The Bayesian estimators ${\hat{\beta }}_{ij}^{*}$ and ${\hat{\mu }}_{ij}^{*}$ are both biased in the frequentist sense, that is, conditional on the true parameter value. This bias is apparent in the two left panels of Figure 3.

The two right panels paint a different picture. They show the bias in the Bayesian sense, that is, conditional on the observed effect. In this sense, the bias is negligible! The small bias that remains is due to our choice of a zero-mean prior while the average of the $b_{ij}^{*}$ is slightly negative.

**Figure 4.**
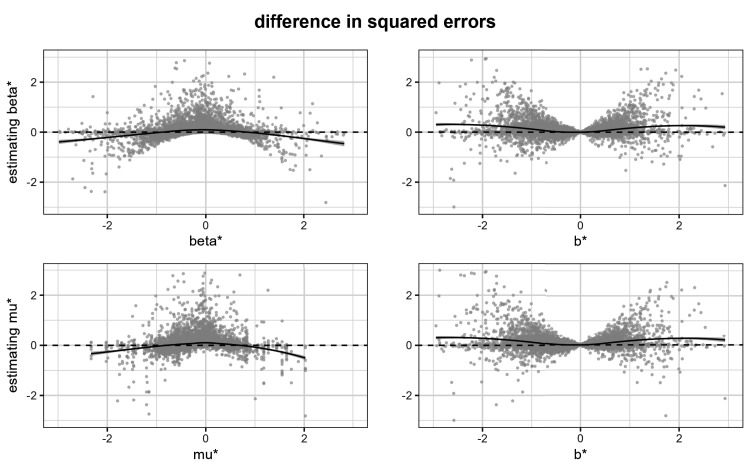
Difference in squared error of the naive and Bayesian estimators. Top row: difference in squared errors for $\beta_{ij}^{*}$ from (8). Bottom row: difference in squared errors for $\mu_{i}^{*}$ , from (9). The two panels on the left side show the frequentist perspective, while the two panels on the right side show the Bayesian perspective.

Figure 4 shows the difference of the squared errors between the naive and Bayesian estimators. To be specific, in the top row we show these differences for estimating the $\beta_{ij}^{*}$

(8) $$(b_{ij}^{*}-\beta_{ij}^{*}{)}^{2}-({\hat{\beta }}_{ij}^{*}-\beta_{ij}^{*}{)}^{2}$$

and in the bottom row we show them for estimating the $\mu_{i}^{*}$

(9) $$(b_{ij}^{*}-\mu_{i}^{*}{)}^{2}-({\hat{\mu }}_{ij}^{*}-\mu_{i}^{*}{)}^{2}$$

In both cases, positive values favor the Bayesian estimators. Again, we added the loess regression curves, which now estimate the following conditional expectations:

$$\begin{array}{rl} & E((b_{ij}^{*}-\beta_{ij}^{*}{)}^{2}-({\hat{\beta }}_{ij}^{*}-\beta_{ij}^{*}{)}^{2}|\beta_{ij}^{*})\;\text{(top left panel of Figure 4)}\\ & E((b_{ij}^{*}-\mu_{i}^{*}{)}^{2}-({\hat{\mu }}_{ij}^{*}-\mu_{i}^{*}{)}^{2}|\mu_{i}^{*})\;\text{(bottom left panel of Figure 4)}\\ & E((b_{ij}^{*}-\beta_{ij}^{*}{)}^{2}-({\hat{\beta }}_{ij}^{*}-\beta_{ij}^{*}{)}^{2}|b_{ij}^{*})\;\text{(top right panel of Figure 4)}\\ & E((b_{ij}^{*}-\mu_{i}^{*}{)}^{2}-({\hat{\mu }}_{ij}^{*}-\mu_{i}^{*}{)}^{2}|b_{ij}^{*})\;\text{(bottom right panel of Figure 4)} \end{array}$$

The two panels on the left side of Figure 4 show the frequentist perspective, where we condition on the true parameter values. We see that the Bayesian estimator performs better for small values of the parameter, while the naive estimator performs better for large values. The two panels on the right side of Figure 4 show the Bayesian perspective, where we condition on the observed $b_{ij}^{*}$ . Unsurprisingly, from the Bayesian perspective, the Bayesian estimators always perform better.

### 3.5. Cross-validation

We have done our best to make sure that the synthetic CDSR closely resembles the true CDSR, so that the results of the previous sections also apply to the true CDSR. However, we cannot exclude the possibility that there are some systematic differences between the synthetic and true datasets which affect the relative performance of the Bayesian and naive estimators. However, we can conduct some additional checks that do not require synthetic CDSR.

First, since the estimates $b_{ij}$ are unbiased for the $\beta_{ij}$ the RMSE is approximately $\sqrt{\frac{1}{18,368}\sum_{ij}s_{ij}^{2}}=0.38$ , which agrees with the value of 0.38 from Table 2. The $b_{ij}$ are also unbiased for the $\mu_{i}$ , so the RMSE can be estimated by $\sqrt{\frac{1}{18,368}\sum_{ij}s_{ij}^{2}+\tau_{i,\text{mle}}^{2}}=0.50$ , which agrees reasonably well with the value of 0.53 in Table 3.

Second, the first column in Table 3 provides an approximation of the difference in mean squared errors across the CDSR,

(10) $$\frac{1}{N}\sum_{ij}(b_{ij}-\mu_{i}{)}^{2}-({\hat{\beta }}_{ij}-\mu_{i}{)}^{2}$$

by the difference across the synthetic CDSR,

(11) $$\frac{1}{N}\sum_{ij}(b_{ij}^{*}-{\hat{\mu }}_{i}{)}^{2}-({\hat{\beta }}_{ij}^{*}-{\hat{\mu }}_{i}{)}^{2}=0.28-0.12=0.16$$

Despite $\mu_{i}$ not being observed, there is an alternative, direct way to estimate (10). We start by constructing a third estimator of $\mu_{i}$ which is unbiased and independent of both $b_{ij}$ and ${\hat{\mu }}_{ij}$ . We leave out study j from meta-analysis i and run a simple fixed effects meta-analysis on the remaining $n_{i}-1$ studies to obtain an estimate ${\hat{\mu }}_{i}^{-j}$ of the population average effect $\mu_{i}$ . This estimator is unbiased for $\mu_{i}$ because the individual study estimates are. Moreover, it is independent of both $b_{ij}$ and ${\hat{\mu }}_{ij}$ because it is based on different studies. According to van Zwet et al.,^11^ we proved the following proposition.

Proposition 1 Consider three estimators $T_{0}$ , $T_{1}$ , and $T_{2}$ of a parameter θ . Suppose that, conditionally on θ , $T_{0}$ is unbiased and independent of $T_{1}$ and $T_{2}$ . Then

(12) $$E(T_{1}-T_{0}{)}^{2}-E(T_{2}-T_{0}{)}^{2}=E(T_{1}-\theta {)}^{2}-E(T_{2}-\theta {)}^{2}$$

where the expectations are with respect to arbitrary distributions of $T_{0}$ , $T_{1}$ , $T_{2}$ , and θ (as long as the expectations are well-defined and finite).

If $T_{0}={\hat{\mu }}_{i}^{-j}$ , $T_{1}=b_{ij}$ , $T_{2}={\hat{\beta }}_{ij}$ , and $\theta =\mu_{i}$ , then it follows that (as N approaches infinity)

(13) $$\frac{1}{N}\sum_{ij}(b_{ij}-\mu_{i}{)}^{2}-({\hat{\beta }}_{ij}-\mu_{i}{)}^{2}\approx \frac{1}{N}\sum_{ij}(b_{ij}-{\hat{\mu }}_{i}^{-j}{)}^{2}-({\hat{\beta }}_{ij}-{\hat{\mu }}_{i}^{-j}{)}^{2}$$

We can compute the right side directly from the original CDSR. We find that it equals 0.19. This is reasonably close to the difference of 0.16 from Table 3. This strengthens our confidence in the results from the synthetic CDSR.

## 4. Discussion

### 4.1. Using these results in applied research

Between-study variation of the treatment effect is often present in systematic reviews. Such heterogeneity may be due to differences in study populations, methodologies, or measurement techniques. In a random effects, meta-analysis the uncertainty due to between-study variation can be accounted for, but it remains hidden when we have only a single trial. In that case, without external information, there is no choice but to identify the within-study effect with the population-level effect. This leads to underestimation of the uncertainty about the population-level effect.

We propose a Bayesian approach, which we refer to as a “meta-analysis of a single trial,” where we estimate the distribution of treatment effects and heterogeneity across 1635 meta-analyses from the CDSR. Taking these estimated distributions as prior information provides a substantial improvement in performance both for estimating the effect in the trial (Table 2) and for estimating the population average effect among similar trials (Table 3). The Bayesian meta-analysis of a single trial can easily be done in R by using package baggr.^6^

The Bayesian approach results in a large reduction of the RMSE across the trials of the CDSR compared to the usual unbiased estimator. This is to be expected for shrinkage estimation, and we have previously obtained similar results.^11^ Here, we want to draw special attention to the substantial lack of coverage of the usual confidence interval for the population average effect; see Table 3. This is due to failure to account for the heterogeneity. In contrast, the coverage of the Bayesian uncertainty interval is equal to its nominal level.

Figure 1 shows that a single trial essentially never provides certainty about the sign of population average effect. This is a strong argument for the need for replication studies.

Since our prior distributions refer to the population of trials in the CDSR, our posterior statements can be interpreted in terms of random sampling from the CDSR. So, for example, we can say that if we randomly select a trial from the CDSR (or from the population of all the trials that could be in the CDSR) and we observe that the estimated treatment effect is b=0.7 with standard error 0.7, then the probability that the true effect in that trial is also positive is 72% (cf. Section 2.2).

Now suppose we are interested in a *particular* trial where the estimated treatment effect is b=0.7 with standard error 0.7. To which extent does that probability of 72% apply? The trial has not been randomly selected from the CDSR. By considering it as such, we are essentially ignoring all of its distinguishing features. An assessment will have to be made if those features should substantially change the 72% probability.

We stress that we do not propose that our method should replace the usual estimate and its standard error. It is important that those are reported so that they may be combined with other information such as a meta-analysis at some later time.

About 75% of the meta-analyses in the CDSR have five or fewer studies.^4^ When a meta-analysis consists of so few studies, it is clear that the heterogeneity cannot be estimated reliably without additional information.^1^ Similarly as in the case of a single study that we outlined here, we should expect that doing a Bayesian meta-analysis with informative priors will improve inference. We intend to further evaluate the performance of this approach in a separate study.

The method we propose is especially well suited to decision making. Consider a decision maker choosing between interventions on the basis of a meta-analysis of a single or just a few studies. A model example would be the health technology assessment (HTA) process, which uses meta-analytic estimates to calculate cost-effectiveness. Both priors we propose play a role here. First, the prior on the mean effect induces shrinkage which helps avoid exaggeration of effect sizes. This leads to better rank ordering of treatments. Next, the prior on the heterogeneity improves the predictive distributions of effects, which can then be used by the decision maker to calculate fully Bayesian estimates of cost effectiveness. This matches the approach implemented in, for example, the UK by NICE (Dias et al.^12^).

### 4.2. Bayesian meta-analysis

Statistical practice—Bayesian and otherwise—has incoherence with respect to the number of studies K in a meta-analysis or, more generally, the number of groups in a multilevel model. We can see this by starting with a large K and then seeing what happens as it decreases.

When K is large, say larger than 10, the study-level variance and thus the optimal shrinkage factor can be well estimated from the data, or if the meta-analysis includes individual and study-level predictors, the unexplained group-level variance can be well estimated.

As K becomes smaller, there is more uncertainty in the study-level variance parameter, and prior information on that parameter becomes more relevant to determining the amount of shrinkage. When K is between 5 and 10, the prior on τ can make more of a difference: first by providing area-specific prior information and second through the regularization properties of weakly informative priors that (probabilistically) constrain the low and high ends of the distribution. A regularizing prior on the high end can be necessary to reduce the upper tail of the posterior for τ ; in full Bayesian inference with a flat prior, the resulting long tail manifests itself by giving some probability of essentially no shrinkage, leading to wide uncertainties for the effects in individual studies. If τ is estimated using a marginal posterior mode, it can also be helpful to use zero-avoiding priors^13^ as otherwise the point estimate for τ can be noisy (zero in some cases and high in others), leading to meta-analyses that uncontrollably swing between complete pooling and little shrinkage in otherwise similar cases. Another advantage of an informative prior is that it reduces the influence of one or two outlying studies.

When K is small, between 3 and 5, it is still possible to perform Bayesian inference on τ with a flat prior, but the resulting posterior mode is noisy and the full Bayesian posterior for τ will have a long right tail,^14^ and so in practice an informative prior for the group-level variance is necessary to avoid the meta-analysis procedure yielding unreasonable results. Setting τ equal to zero to perform a so-called fixed or common effects meta-analysis amounts to using a extremely strong and unrealistic prior.

With K=2 , the mathematical situation changes: a flat prior on τ yields an improper posterior distribution with an infinite right tail. This is related to the result from James-Stein theory that the no-shrinkage estimate is admissible when K<3 . In practice, though, there is no sharp boundary between two and three studies, as in either case we want to be using a strong prior for τ . At K=2 , we should expect the prior for τ to dominate to the extent that the amount of shrinkage is determined much more by the prior—that is, by the population of studies being considered as the reference class—than by the observed spread in the data.

When K=1 , the problem doesn’t look like meta-analysis at all: it is just inference from a single study. This leads to the paradox that removing information can be expected to decrease reported uncertainty. The paradox is resolved by recognizing that the estimand has changed. The solution is to consider questions of meta-analysis and between-study variation even in a single study. In other words, this means placing the problem in a hierarchical context: even when multiple data sources are not available, we can still include a prior on between-study variation. This was already going to be necessary with K=2 or 3, so why not do this with K=1 also?

Finally, K=0 corresponds to the setting where no studies are available on a problem of interest, so that the posterior is determined entirely by the prior. This can be viewed as a sort of thought experiment, representing the information being assumed from nothing but the general class of problems under study.

## 5. Reproducibility

The results in this article are fully reproducible with the R code provided in GitHub repository at github.com/wwiecek/singletrial. The data are publicly available at https://osf.io/xjv9g/. Below we provide the code snippet for calculating probability of positive effects in a trial or population; see the example in Section 2.2.


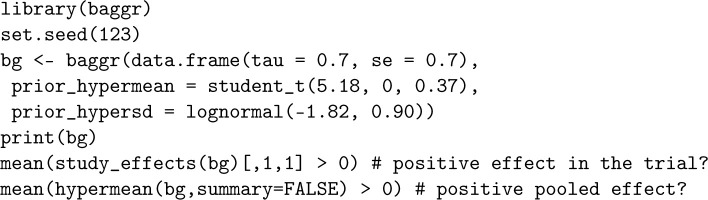


## Supplemental Material

sj-pdf-1-smm-10.1177_09622802251380628 - Supplemental material for Meta-analysis with a single study Supplemental material, sj-pdf-1-smm-10.1177_09622802251380628 for Meta-analysis with a single study by Erik van Zwet, Witold Wiȩcek and Andrew Gelman in Statistical Methods in Medical Research
