# Supplementary material for: Meta-analysis with a single study
Source: Stat Methods Med Res. 2025 Oct 9;34(12):2302–12. doi: 10.1177/09622802251380628 (PMC12669397; doi:10.1177/09622802251380628)
Supplement: sj-pdf-1-smm-10.1177_09622802251380628 - Supplemental material for Meta-analysis with a single study [file sj-pdf-1-smm-10.1177_09622802251380628.pdf]

Meta-analysis with a single study - supplement

Erik van Zwet, Witold Więcek, Andrew Gelman

02 December, 2024

Contents

1 Packages and utility functions **2**

2 Data **2**

3 Leave-one-out validation **4**

3.1 Estimate the distribution of mu and tau; zero mean 4

3.2 Leave-one-out meta-analyses 6

3.3 Single study meta-analyses on original data 6

3.4 Difference in MSE 6

3.5 Probabilities 7

4 Synthetic CDSR **8**

4.1 Estimate mu and tau2 (for ordering) 8

4.2 Estimate the distribution of mu and tau; non-zero mean 8

4.3 Synthetic CDSR 11

4.4 Single study meta-analyses on synthetic data 13

4.5 Evaluate and compare 14

4.5.1 Probabilities 14

4.5.2 Tables 15

5 Frequentist perspective **18**

5.1 Bias 18

5.2 Difference in MSE 19

This PDF supplement was generated using Rmarkdown in R.

All code is available at `https://github.com/wwiecek/singletrial`.

1 Packages and utility functions

```
# packages and helper functions
suppressPackageStartupMessages({
  library(dplyr)
  library(tidyr)
  library(ggplot2)
  library(cowplot)
  library(kableExtra)
  library(xtable)
  #library(splines)
  library(scam)      # for monotone regression
  library(mgcv)      # gam with te()
  #library(quantreg)  # for quantile regression rqs()
  library(metafor)
  library(baggr)
  library(rstan)
})

options(mc.cores = 4)

# generalized t distribution
mydt = function(x, m, s, df) dt((x-m)/s, df)/s   # generalized t distribution


dmix = function(x,p,m,s){                        # normal mixture distribution
  p %*% sapply(x, function(x) dnorm(x,mean=m,sd=s))
}

rmix = function(n,p,m,s){                        # sample from normal mixture
  d=rmultinom(n,1,p)
  rnorm(n,m%*%d,s%*%d)
}

mydt = function(x, m, s, df) dt((x-m)/s, df)/s  # generalized t density
```

2 Data

In Nov 2024 we realised that a logit transform was making binary event studies non-comparable to continuous outcome studies, so we swapped it for probit Below we show the contents of a new data wrangling script, `prepare_cdsr_data.R`, which we used to generate the transformed dataset, i.e. one in which size effects are calculated from group means or event numbers

```
set.seed(123)

load("data/CDSR.RData")  # read dataframe "data"

d = dplyr::filter(data, outcome.flag %in% c("CONT","DICH") &
             outcome.group=="efficacy" &
             outcome.nr==1 &
             comparison.nr==1)
```

```r
# make sure each study used only once:
d=group_by(d,study.name) %>% sample_n(size=1)

d1=dplyr::filter(d, outcome.flag=="CONT")
d1=escalc(m1i=mean1,sd1i=sd1,n1i=total1,
          m2i=mean2,sd2i=sd2,n2i=total2,measure="SMD",
          data=d1, append=TRUE)
d2=dplyr::filter(d, outcome.flag=="DICH")
d2=escalc(ai=events1,n1i=total1,
          ci=events2,n2i=total2,measure="PBIT",
          data=d2,append=TRUE)
d=rbind(d1,d2)

d=mutate(d,b=yi,s=sqrt(vi),z=b/s)
d=dplyr::filter(d,abs(z)<20, abs(b)<5)

d$b=d$yi
d$s=sqrt(d$vi)
d$z=d$b/d$s

saveRDS(d, file = "transformed_data/cdsr_smd_pbit.rds")
```

Read the transformed CDSR data and put into data.frame `d`. Count studies per meta-analysis in data.frame `meta`.

```r
d <- readRDS("transformed_data/cdsr_smd_pbit.rds")

d=arrange(d,id)
d=group_by(d,id) %>% mutate(k = n()) %>% ungroup()   # count studies within meta-analysis
meta=group_by(d,id) %>% summarise(k = first(k))
mean(meta$k == 1)
```

```
## [1] 0.3145877
```

```r
mean(meta$k >= 20)
```

```
## [1] 0.03113795
```

```r
mean(meta$k <= 5)
```

```
## [1] 0.750519
```

That 75% is the same as Davey J, Turner RM, Clarke MJ, Higgins JPT. Characteristics of meta-analyses and their component studies in the Cochrane Database of Systematic Reviews: a cross-sectional, descriptive analysis. BMC Medical Research Methodology 2011; 11:160.

```r
median(meta$k[meta$k > 1])
```

```
## [1] 4
```

Select meta-analyses with at least 5 studies.

```r
d=filter(d,k>4)                          # at least 5 studies
n_meta=length(unique(d$id))              # total number of meta-analyses
n_meta
```

```
## [1] 1635
```

```r
n_trials=nrow(d)
n_trials                                 # total number of trials
```

```
## [1] 18368
```

```r
d=droplevels(d)
```

3 Leave-one-out validation

3.1 Estimate the distribution of mu and tau; zero mean

The prior for the inverse of the number of degrees of freedom is from page 372 of Advanced Regression and Multilevel Models.

```r
model = 'data {
  int<lower=0>N; // number of trials
  int<lower=0>k; // number of meta-analyses
  int<lower=0>n[k]; // number of trials per meta-anlysis
  int<lower=0>begin[k];
  int<lower=0>end[k];
  vector[N] b; // estimates
  vector[N] se2; // squared standard errors of b
}
parameters {
  real<lower=0.02, upper=0.5> invnu;       // inverse degrees of freedom of mu
  real<lower=-1, upper=-0.5> log_s_mu;     // log scale of mu
  real<lower=-0.5, upper=0> log_s_logtau;  // log scale of logtau
  real<lower=-2, upper=-1> m_logtau;       // mean of logtau
  vector[k] mu;                            // mean effects in meta-analyses
  vector[k] logtau;                        // within-meta-analysis heterogeneities
}
transformed parameters{
  real<lower=0> nu=1/invnu;
  real s_mu=exp(log_s_mu);
  real s_logtau=exp(log_s_logtau);
  vector<lower=0>[k] tau=exp(logtau);
  vector<lower=0>[k] tau2=tau^2;
}
model {
  mu ~ student_t(nu, 0, s_mu);
  logtau ~ normal(m_logtau,s_logtau);
  for (i in 1:k){
    target += normal_lpdf(b[begin[i]:end[i]] | mu[i],
    sqrt(se2[begin[i]:end[i]] + tau2[i]));
```

```
  }
}'

n=d %>% group_by(id) %>% summarise(n = n())
n=n$n
k=length(n) # number of meta-analyses
begin=cumsum(c(1,n[1:(k-1)]))
end=cumsum(n)
dat=list(n=n,N=length(d$b),k=k,b=d$b,se2=d$s^2,begin=begin,end=end) # data for Stan

m = stan_model(model_code=model)
fit0=sampling(object=m,
              data=dat,warmup=2000,iter=4000,chains=4,refresh=0,
              pars = c("invnu","log_s_mu", "log_s_logtau",
                       "m_logtau", "nu","s_mu","s_logtau"), include = TRUE)

save(fit0, file="results/cdsr_fit_zeromean.Rdata")
```

```
load("results/cdsr_fit_zeromean.Rdata")
print(traceplot(fit0,c("invnu","log_s_mu",
                       "log_s_logtau",
                       "m_logtau"),inc_warmup=TRUE))
```

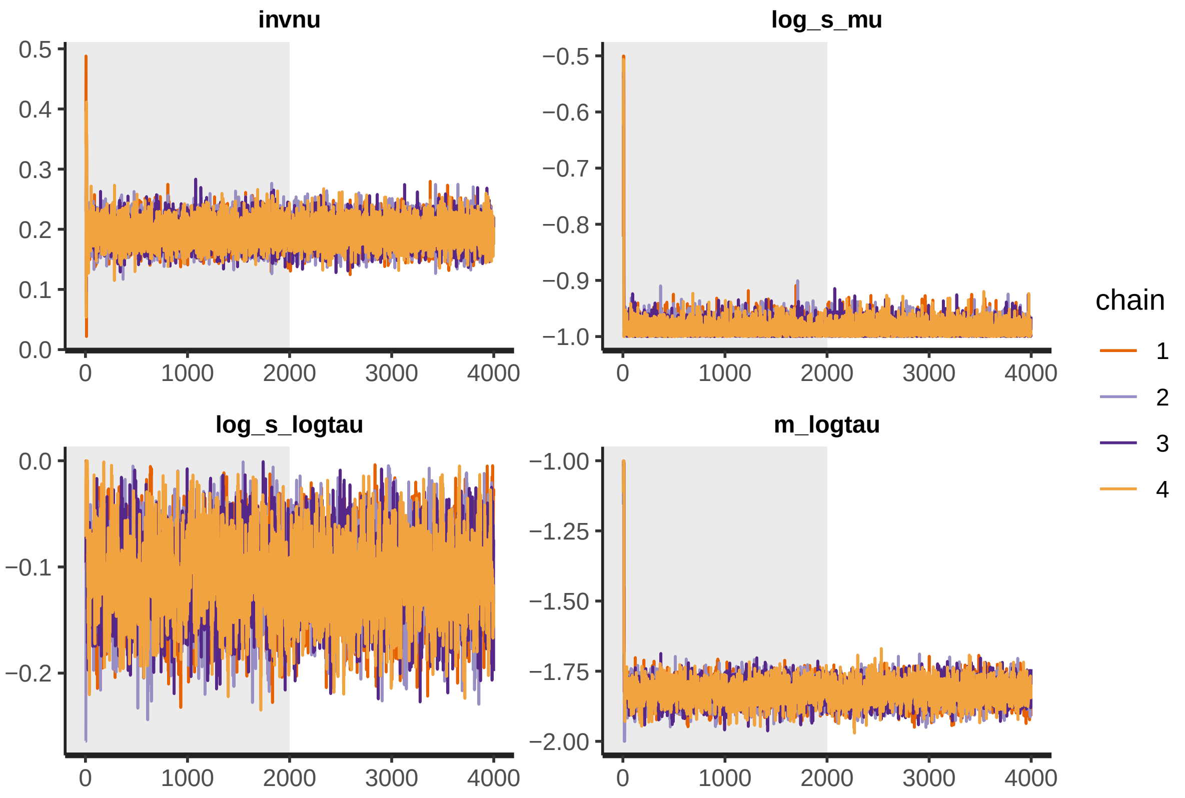


```
tmp=summary(fit0, pars = c("nu","s_mu","s_logtau","m_logtau"),
            probs = c(0.025, 0.975))$summary
print(tmp)
```

```
##                  mean      se_mean          sd        2.5%       97.5%      n_eff
```

```
## nu        5.1752702 5.477667e-03 0.57936312  4.2145785  6.4838414 11186.916
## s_mu      0.3725950 4.156494e-05 0.00431742  0.3680052  0.3838928 10789.325
## s_logtau  0.8978851 9.304264e-04 0.03274222  0.8352530  0.9634679  1238.375
## m_logtau -1.8248905 9.528799e-04 0.03853633 -1.9011270 -1.7501472  1635.552
##               Rhat
## nu       0.9997138
## s_mu     0.9999792
## s_logtau 1.0022147
## m_logtau 1.0027897
```

```
# Values before we switched to probit from logit, for future reference:
# distr0=data.frame(m_mu=0,s_mu=0.48,nu=3.8,m_logtau=-1.44,s_logtau=0.79)

distr0 <- c(m_mu = 0, apply(as.matrix(fit0), 2, mean)[c("s_mu", "nu", "m_logtau", "s_logtau")])
```

3.2 Leave-one-out meta-analyses

Run all leave-one-out fixed effects meta-analyses and add `mu.loo` to data.frame `data`

3.3 Single study meta-analyses on original data

```
load("results/d.Rdata")
options(mc.cores = NULL)
N=nrow(d)
for (i in 1:N){
  # cat("\r",i," out of",N)
  fit=baggr(data.frame(tau=d$b[i],se=d$s[i]),model="rubin",
            prior_hypermean=student_t(distr0[["nu"]], distr0[["m_mu"]], distr0[["s_mu"]]),
            prior_hypersd=lognormal(distr0[["m_logtau"]], distr0[["s_logtau"]]),
            chains=4,refresh=0)
  stanfit=fit$fit                            # stanfit object
  d$Rhat[i]=max(summary(stanfit)$summary[,10])
  if (d$Rhat[i] > 1.01){
    fit=baggr(data.frame(tau=d$b[i],se=d$s[i]),model="rubin",
              prior_hypermean=student_t(distr0[["nu"]], distr0[["m_mu"]], distr0[["s_mu"]]),
              prior_hypersd=lognormal(distr0[["m_logtau"]], distr0[["s_logtau"]]),
              warmup=2000,iter=5000,chains=4,refresh=0)
    stanfit=fit$fit
    d$Rhat[i]=max(summary(stanfit)$summary[,10])
  }
  draws = as.data.frame(stanfit)             # get posterior draws
  summ=summary(stanfit)$summary
  d$muhat[i]=summ[1,1]             # pooled effect in meta-analysis
  d$p1[i]=mean(d$b[i]*draws$"theta_k[1]" > 0)  # sign in the trial
  d$p2[i]=mean(d$b[i]*draws$"mu[1]" > 0)    # sign of pooled effect
}
save(d,file="results/d.Rdata")  # data.frame "d"
```

3.4 Difference in MSE

Differences of MSE in real CDSR via leave-one-out trick

```r
load(file="results/d.Rdata")   # data.frame "d"
mean((d$mu.loo - d$b)^2 - (d$mu.loo - d$muhat)^2)
```

```
## [1] 0.1936267
```

3.5 Probabilities

Plot the 2 relevant probabilities of the correct sign, and fit smooth regression curves.

```r
probs=d[,c("z","p1","p2")] %>% pivot_longer(cols=c("p1","p2"))

lab1=as.character(expression(paste("P(",b %*% beta > 0," | |z|)")))
lab2=as.character(expression(paste("P(",b %*% mu > 0," | |z|)")))

cbPalette <- c("#E69F00", "#56B4E9")

ggplot(probs,aes(x=abs(z),y=value,group=name,color=name)) +
  geom_point(size=0.1,alpha=0.1) +
  geom_smooth(method = "gam",
              formula = y ~ s(x, k = 10),
              se = FALSE,linewidth=0.5,color="black") +
  annotate("text",x = 5.2, y = 1.03, label = lab1, parse=TRUE, hjust=0) +
  annotate("text",x = 5.2, y = 0.9, label = lab2, parse=TRUE, hjust=0) +
  scale_y_continuous(limits = c(0.5, 1.05), breaks = seq(0.5, 1, by = 0.1)) +
  scale_x_continuous(limits = c(0, 8), breaks = seq(0, 5, by = 1),
                     minor_breaks= seq(0, 5, by = 0.5)) +
  scale_colour_manual(values=cbPalette) +
  xlab("|z-value|") + ylab("probability") +
  guides(color="none") + theme_bw()
```

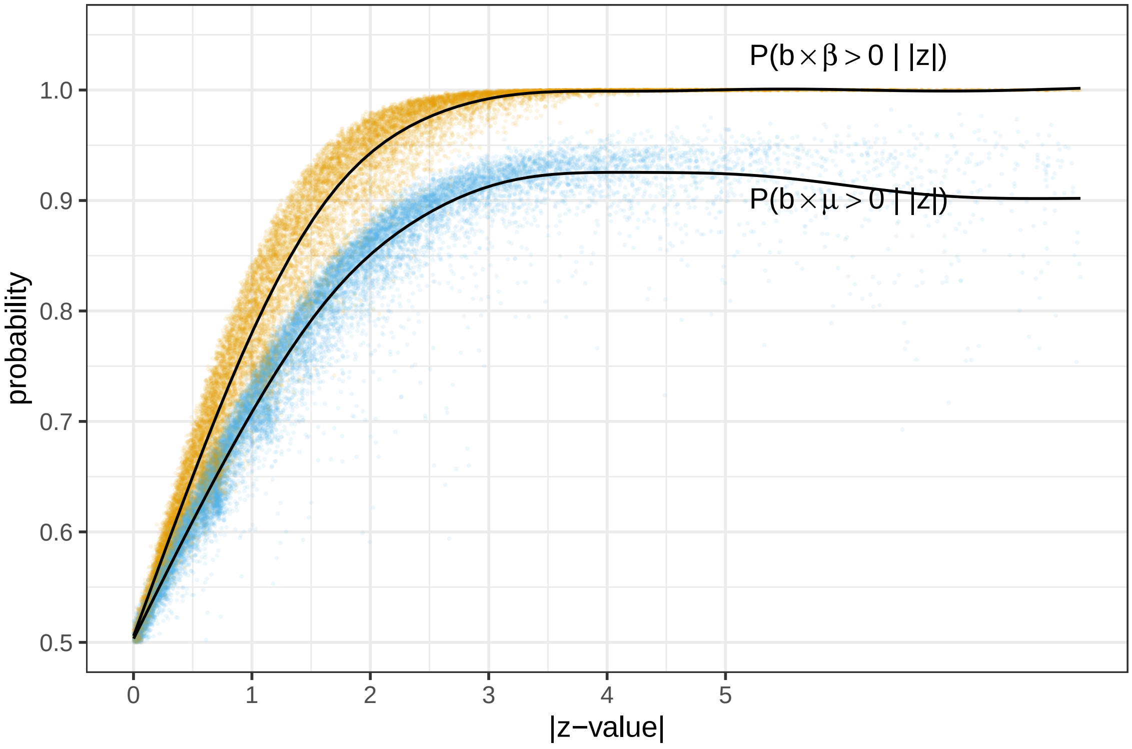

```
ggsave("figures/probs.pdf")
ggsave("figures/probs.png",dpi=300)
```

4 Synthetic CDSR

4.1 Estimate mu and tau2 (for ordering)

Run meta-analyses to estimate *mu* and τ^2. Only their ordering is used in the construction of the synthetic CDSR. We use the default of `metafor::rma`.

```
ma = function(b,s){          # perform meta-analyses
  out = tryCatch(
    { fit=rma(yi=b,sei=s, method="ML",
              control=list(maxiter = 10000,stepadj=0.5))
    mu=drop(fit$beta)
    tau=sqrt(fit$tau2)
    },
    error=function(cond) {
      mu=NA
      tau=NA
    }
  )
  return(data.frame(mu=mu,tau=tau))
}
d=d %>% group_by(id) %>% mutate(ma(b,s)) %>% ungroup()
n=nrow(d)
d$tau=d$tau + runif(n,-10^(-8),10^(-8))  # jitter
save(d,file="results/d.Rdata")
```

We can now directly estimate the MSE of b as an (unbiased) estimator of β and μ

```
mean(d$s^2)             # MSE for estimating beta
```

```
## [1] 0.1448569
```

```
mean(d$s^2 + d$tau^2)   # MSE for estimating mu
```

```
## [1] 0.2460857
```

```
sqrt(mean(d$s^2))             # RMSE for estimating beta
```

```
## [1] 0.3806007
```

```
sqrt(mean(d$s^2 + d$tau^2))   # RMSE for estimating mu
```

```
## [1] 0.4960703
```

4.2 Estimate the distribution of mu and tau; non-zero mean

```
model = 'data {
  int<lower=0>N; // number of trials
  int<lower=0>k; // number of meta-analyses
  int<lower=0>n[k]; // number of trials per meta-anlysis
  int<lower=0>begin[k];
  int<lower=0>end[k];
  vector[N] b; // estimates
  vector[N] se2; // squared standard errors of b
}
parameters {
  real<lower=0.02, upper=0.5> invnu;       // inverse degrees of freedom of mu
  real<lower=-0.5, upper=0> m_mu;          // mean of mu
  real<lower=-1, upper=-0.5> log_s_mu;     // log scale of mu
  real<lower=-0.5, upper=0> log_s_logtau;  // log scale of logtau
  real<lower=-2, upper=-1> m_logtau;       // mean of logtau
  vector[k] mu;                            // mean effects in meta-analyses
  vector[k] logtau;                        // within-meta-analysis heterogeneities
}
transformed parameters{
  real<lower=0> nu=1/invnu;
  real s_mu=exp(log_s_mu);
  real s_logtau=exp(log_s_logtau);
  vector<lower=0>[k] tau=exp(logtau);
  vector<lower=0>[k] tau2=tau^2;
}
model {
  invnu ~ uniform(0.02, 0.5); // page 372 of Advanced Regression and Multilevel Models
  m_logtau ~ normal(0,2);
  mu ~ student_t(nu, m_mu, s_mu);
  m_logtau ~ normal(0,4);
  logtau ~ normal(m_logtau,s_logtau);
  for (i in 1:k){
    target += normal_lpdf(b[begin[i]:end[i]] | mu[i],
    sqrt(se2[begin[i]:end[i]] + tau2[i]));
}
}'

n=d %>% group_by(id) %>% summarise(n = n())
n=n$n
k=length(n) # number of meta-analyses
begin=cumsum(c(1,n[1:(k-1)]))
end=cumsum(n)
dat=list(n=n,N=length(d$b),k=k,b=d$b,se2=d$s^2,begin=begin,end=end) # data for Stan
options(mc.cores = 4)
m = stan_model(model_code=model)
fit=sampling(object=m,data=dat,warmup=2000,iter=4000,chains=4,refresh=0,
             pars = c("invnu","m_mu","log_s_mu", "log_s_logtau","m_logtau",
                      "nu","s_mu","s_logtau"))
save(fit,file="results/cdsr_fit_nonzeromean.Rdata")
```

```
load("results/cdsr_fit_nonzeromean.Rdata")
print(traceplot(fit,c("invnu","m_mu","log_s_mu",
                      "log_s_logtau","m_logtau"),inc_warmup=TRUE))
```

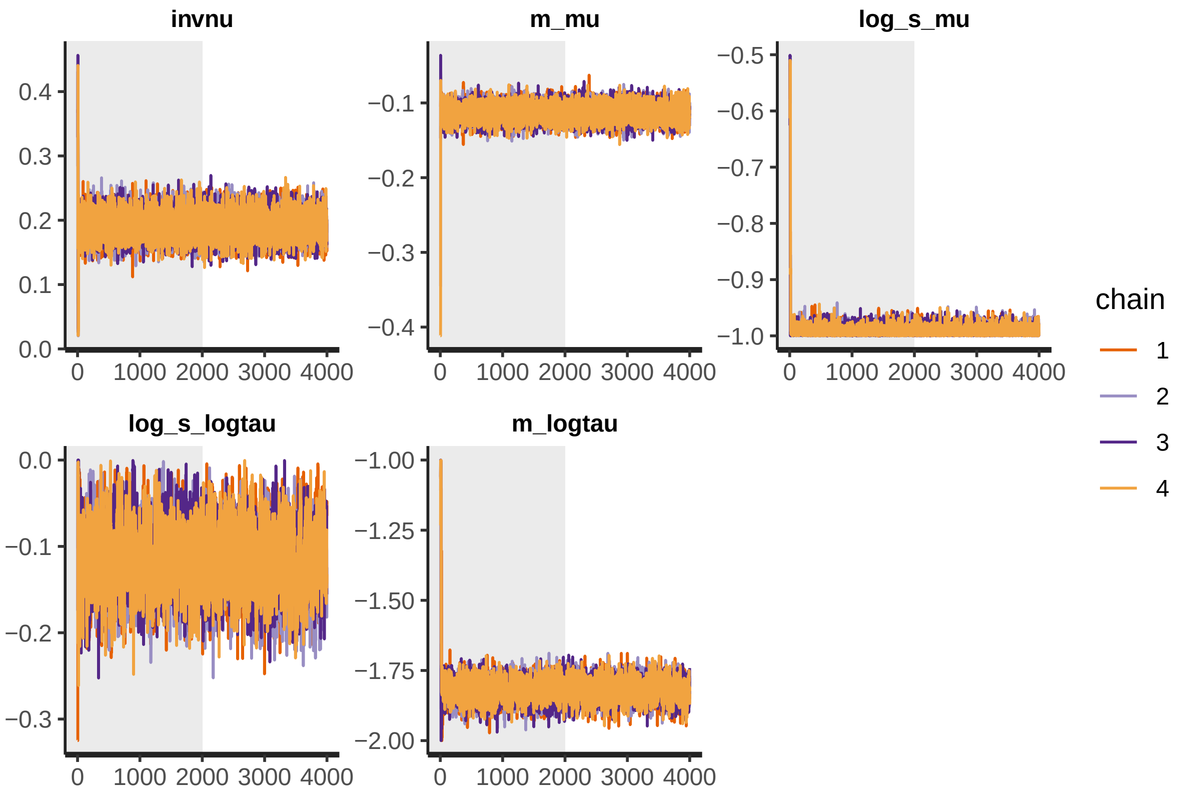


```r
tmp=summary(fit, pars = c("nu","m_mu","s_mu","m_logtau","s_logtau"),
               probs = c(0.025, 0.975))$summary
print(tmp)
```

```
##                 mean      se_mean          sd       2.5%       97.5%      n_eff
## nu         5.2971303 5.722524e-03 0.576622579  4.3047969  6.55739123 10153.3208
## m_mu      -0.1128916 1.001470e-04 0.011142647 -0.1344897 -0.09084236 12379.4397
## s_mu       0.3706504 2.492482e-05 0.002675925  0.3679341  0.37770558 11526.1342
## m_logtau  -1.8191596 1.036534e-03 0.038691626 -1.8971128 -1.74609721  1393.3711
## s_logtau   0.8919663 1.097083e-03 0.033657953  0.8283804  0.95965040   941.2315
##                Rhat
## nu        0.9999758
## m_mu      0.9997294
## s_mu      0.9997730
## m_logtau  1.0038663
## s_logtau  1.0054224
```

```r
# Old result (before using probit instead of logit):
# distr=data.frame(m_mu=-0.17,s_mu=0.43,nu=3.05,m_logtau=-1.44,s_logtau=0.78)

distr_non0 <- c(apply(as.matrix(fit), 2, mean)[c("m_mu", "s_mu", "nu", "m_logtau", "s_logtau")])
distr <- rbind(round(distr0, 2),
               round(distr_non0, 2))

kable(distr)
```

m_mu	s_mu	nu	m_logtau	s_logtau
0.00	0.37	5.18	-1.82	0.90
-0.11	0.37	5.30	-1.82	0.89

```
xtable(distr)
```

```
% latex table generated in R 4.2.2 by xtable 1.8-4 package
% Mon Dec  2 11:37:33 2024
\begin{table}[ht]
\centering
\begin{tabular}{rrrrrr}
  \hline
 & m\_mu & s\_mu & nu & m\_logtau & s\_logtau \\
  \hline
1 & 0.00 & 0.37 & 5.18 & -1.82 & 0.90 \\
  2 & -0.11 & 0.37 & 5.30 & -1.82 & 0.89 \\
   \hline
\end{tabular}
\end{table}
```

4.3 Synthetic CDSR

```
set.seed(123)
n=nrow(d)

# count studies within meta-analysis:
d=group_by(d,id) %>% mutate(k = n()) %>% ungroup()
meta=group_by(d,id) %>% summarise(mu=first(mu),
                                  tau=first(tau),
                                  k = first(k))
N=nrow(meta)
ind=order(meta$mu)
mu=distr_non0[["s_mu"]]*rt(N, distr_non0[["nu"]]) + distr_non0[["m_mu"]]
mu=sort(mu)
mu=mu[order(ind)]
mu=rep(mu,meta$k)

ind=order(meta$tau)
logtau=rnorm(N, distr0[["m_logtau"]], distr0[["s_logtau"]])
logtau=sort(logtau)
logtau=logtau[order(ind)]
logtau=rep(logtau,meta$k)
tau=exp(logtau)

beta=rnorm(n,mu,tau)
b=beta + rnorm(n,0,d$s)
z=b/d$s
z.abs = abs(z)

sim=data.frame(id=d$id,mu,tau,beta,b,s=d$s,z,z.abs)
```

```r
save(sim,file="results/simulated_cdsr.Rdata")
```

Compare synthetic to original

```r
load("results/simulated_cdsr.Rdata")
d1=data.frame(b=d$b,z=d$z,type="original")
d2=data.frame(b=sim$b,z=sim$z,type="simulated")
df=rbind(d1,d2)
p1=ggplot(df, aes(x=b, fill=type)) + geom_density(alpha=.25) +
  scale_fill_manual(values=c("white", "grey40")) +
  xlim(-8,8) + ylab('') + guides(fill="none") + theme_bw()
p2=ggplot(df, aes(x=z, fill=type)) + geom_density(alpha=.25) +
  scale_fill_manual(values=c("white", "grey40"), name="") +
  xlim(-8,8) + ylab('') + theme_bw() +
  theme(legend.position = "bottom")
legend=get_legend(p2)
ggp=plot_grid(p1, p2 + guides(fill="none"), nrow=1,rel_widths = c(1,1))
plot_grid(ggp,legend, nrow=2, rel_heights = c(1,0.1)) +
  theme(plot.caption = element_text(hjust = 0,  size = 9))
```

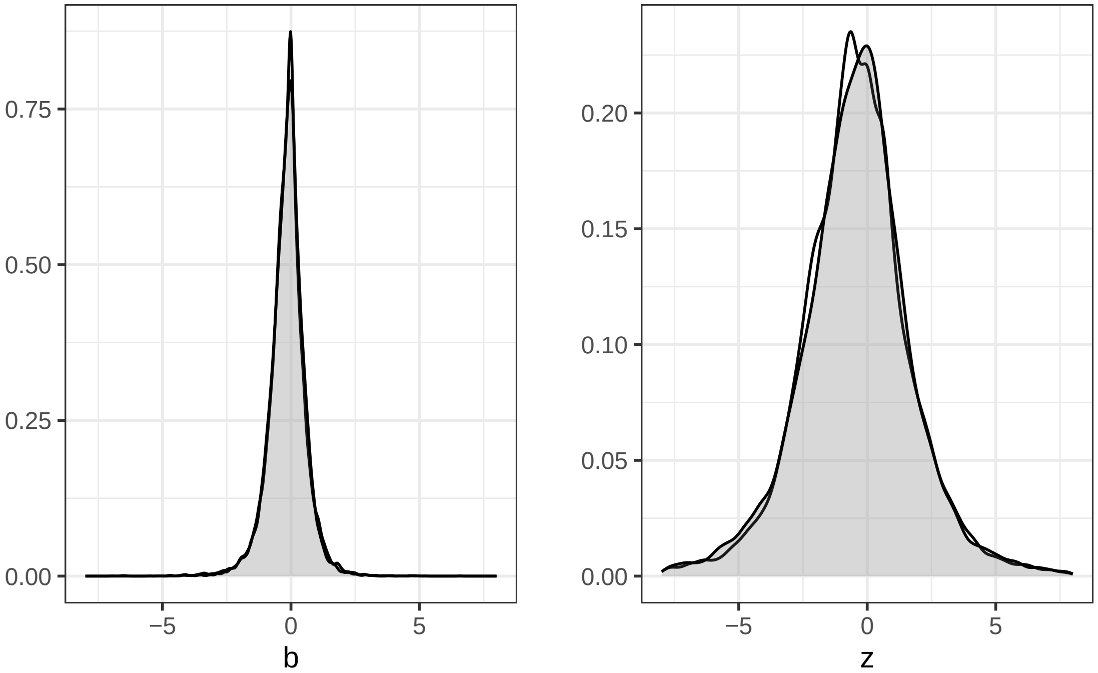


```r
ggsave("figures/synthetic_CDSR.pdf")
```

Make a figure of μ^* versus τ^* to show their dependence.

```r
df=group_by(sim,id) %>% summarise(mu=first(mu),tau=first(tau))
ggplot(df,aes(x=mu,y=tau)) + geom_point(alpha=0.1,size=1) +
  geom_smooth() + xlim(-2.5,2.5) + theme_bw()
```

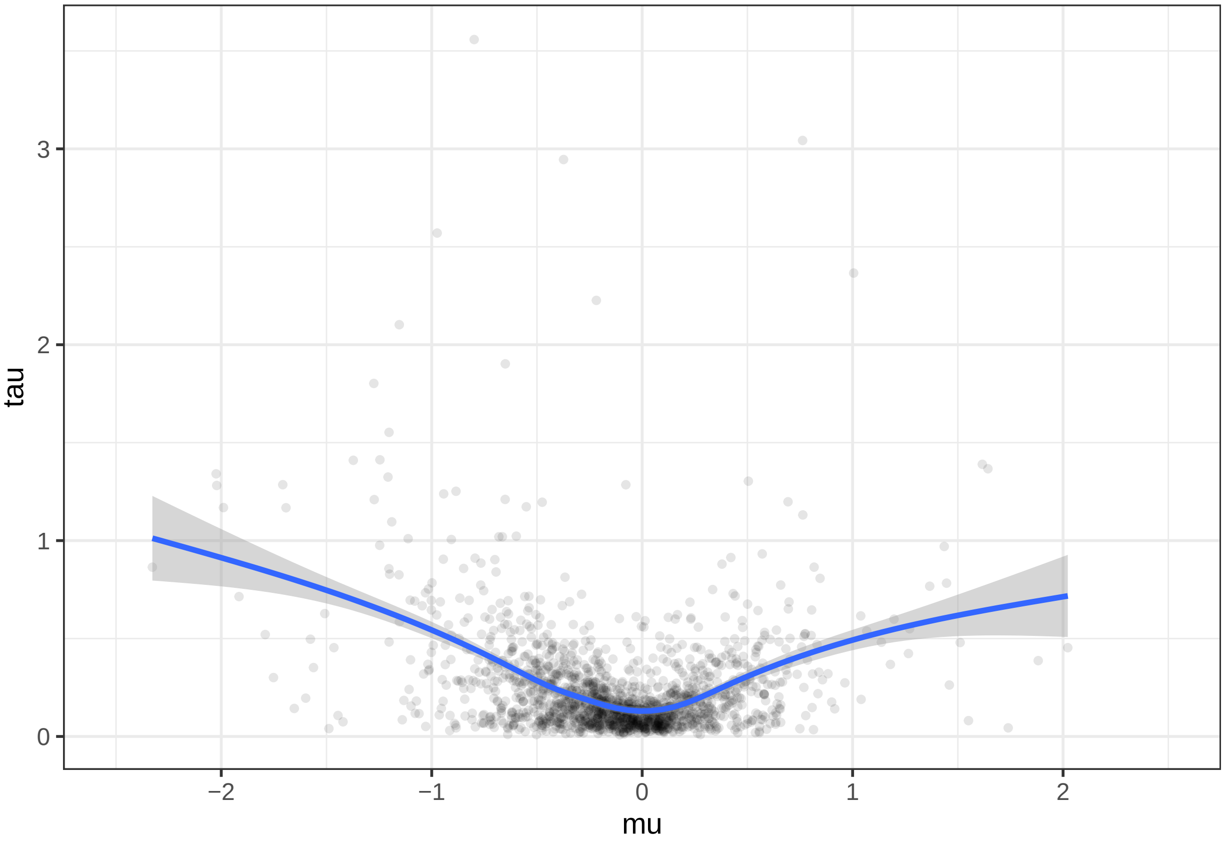

4.4 Single study meta-analyses on synthetic data

```
N=nrow(sim)
for (i in 1:N){
  # cat("\r",i," out of",N)
  fit=baggr(data.frame(tau=sim$b[i],se=sim$s[i]),model="rubin",
            prior_hypermean=student_t(distr0[["nu"]],0,distr0[["s_mu"]]),
            prior_hypersd=lognormal(distr0[["m_logtau"]], distr0[["s_logtau"]]),
            chains=4,refresh=0)
  stanfit=fit$fit                              # stanfit object
  sim$Rhat[i]=max(summary(stanfit)$summary[,10])
  if (sim$Rhat[i] > 1.01){
    fit=baggr(data.frame(tau=sim$b[i],se=sim$s[i]),model="rubin",
              prior_hypermean=student_t(distr0[["nu"]],0,distr0[["s_mu"]]),
              prior_hypersd=lognormal(distr0[["m_logtau"]], distr0[["s_logtau"]]),
              warmup=2000,iter=5000,chains=4,refresh=0)
    stanfit=fit$fit
    sim$Rhat[i]=max(summary(stanfit)$summary[,10])
  }

  draws = as.data.frame(stanfit)               # get posterior draws
  summ=summary(stanfit)$summary

  sim$betahat[i]=summ[4,1]       # effect in trial
  sim$betahat_se[i]=summ[4,3]
```

```r
  sim$betahat_L[i]=summ[4,4]
  sim$betahat_U[i]=summ[4,8]
  sim$p1[i]=mean(sim$b[i]*draws$"theta_k[1]" > 0)

  sim$muhat[i]=summ[1,1]      # pooled effect in meta-analysis
  sim$muhat_se[i]=summ[1,3]
  sim$muhat_L[i]=summ[1,4]
  sim$muhat_U[i]=summ[1,8]
  sim$p2[i]=mean(sim$b[i]*draws$"mu[1]" > 0)
}
save(sim,file="results/simulated_cdsr.Rdata")
```

4.5 Evaluate and compare

4.5.1 Probabilities

Compare the probabilities of the correct sign in the synthetic CDSR to smooth regression models.

```r
load("results/simulated_cdsr.Rdata")
probs=sim[,c("z","p1","p2")] %>% pivot_longer(cols=c("p1","p2"))

lab1=as.character(expression(paste("P(",b %*% beta > 0," | |z|)")))
lab2=as.character(expression(paste("P(",b %*% mu > 0," | |z|)")))

ggp=ggplot(probs,aes(x=abs(z),y=value,group=name)) +
  geom_smooth(method = "gam",
              formula = y ~ s(x, k = 10),
              se = FALSE,size=1,color="lightgrey") +
  annotate("text",x = 5.2, y = 1.03, label = lab1, parse=TRUE, hjust=0) +
  annotate("text",x = 5.2, y = 0.9, label = lab2, parse=TRUE, hjust=0) +
  scale_y_continuous(limits = c(0.5, 1.05), breaks = seq(0.5, 1, by = 0.1)) +
  scale_x_continuous(limits = c(0, 8), breaks = seq(0, 5, by = 1),
                     minor_breaks= seq(0, 5, by = 0.5)) +
  xlab("z-value") + ylab("probability") +
  theme_bw()

fit=gam((b*beta)>0 ~ s(z.abs,k=10),data=sim)
pred <-  predict(fit, newdata=data.frame(z.abs=seq(0,5,0.01)),type="response")
df1=data.frame(z.abs=seq(0,5,0.01),pred=pred,label="beta")

fit=gam((b*mu)>0 ~ s(z.abs,k=10),data=sim)
pred <-  predict(fit, newdata=data.frame(z.abs=seq(0,5,0.01)),type="response")
df2=data.frame(z.abs=seq(0,5,0.01),pred=pred,label="mu")

df=rbind(df1,df2)

ggp + geom_line(data=df,aes(x = z.abs, y = pred, group=label)) +
  theme(plot.caption = element_text(hjust = 0)) +
  labs(caption = "The probability that the sign of the estimated effect matches the sign of the true ef
```

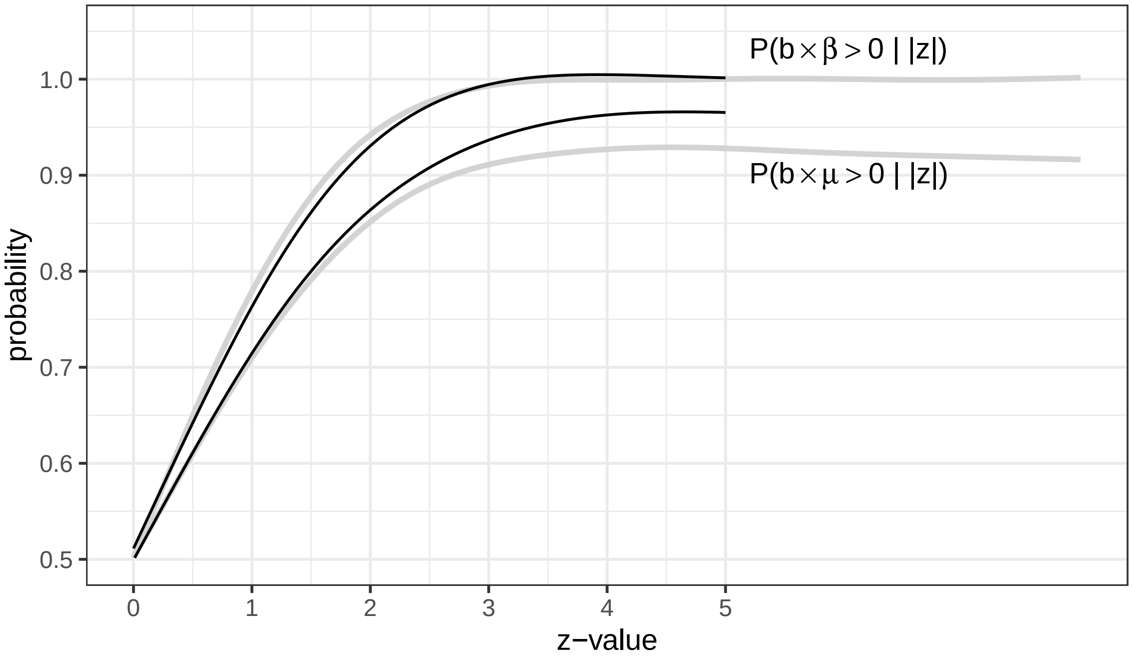

The probability that the sign of the estimated effect matches the sign of the true effect or the average effect among similar trials. The black lines are direct estimates while the grey lines are from the Bayesian analysis.

4.5.2 Tables

```
# sim=sim[sim$Rhat < 1.01,]
ind=which(abs(sim$b/sim$s) > 1.96)
sig=sim[ind,]

maketable=function(par,b,se,est,L,U){
  tab=data.frame(method=c("naive","Bayes"),RMSE=NA,bias=NA,coverage=NA)
  tab$RMSE[1]=sqrt(mean((par - b)^2))
  tab$RMSE[2]=sqrt(mean((par - est)^2))

  tab$bias[1]=mean(abs(b) - abs(par))
  tab$bias[2]=mean(abs(est) - abs(par))

  tab$coverage[1]=mean(abs(par - b) < 1.96*se)
  tab$coverage[2]=mean((L < par) & (U > par))

  tab=tab %>% mutate_if(is.numeric, round, digits=2)
  return(tab)
}

par=sim$beta
b=sim$b
se=sim$s
est=sim$betahat
L=sim$betahat_L
U=sim$betahat_U
```

```r
tab1=maketable(par,b,se,est,L,U)

par=sig$beta
b=sig$b
se=sig$s
est=sig$betahat
L=sig$betahat_L
U=sig$betahat_U
tab2=maketable(par,b,se,est,L,U)

caption="The mean squared error and coverage for estimating $\\beta_i$, i.e. the effect
in the trial. On the right-hand side of the table, we condition
on statistical significance, i.e. $|b_i/s_i| > 1.96$."

tab=cbind(tab1,tab2[2:4])
kable(tab,caption=caption,label="tab:beta") %>%
  add_header_above(header=c(" "=1,"all"=2,"significant only"=4)) %>%
  kable_styling(latex_options = "HOLD_position")
```

4.5.2.1 Effect in the study

Table 2: The mean squared error and coverage for estimating β_i, i.e. the effect in the trial. On the right-hand side of the table, we condition on statistical significance, i.e. $|b_i/s_i| > 1.96$.

	all		significant only			
method	RMSE	bias	coverage	RMSE	bias	coverage
naive	0.38	0.10	0.95	0.42	0.21	0.90
Bayes	0.29	-0.06	0.95	0.29	-0.01	0.95

```r
xtable(tab)
```

```
% latex table generated in R 4.2.2 by xtable 1.8-4 package
% Mon Dec  2 11:37:39 2024
\begin{table}[ht]
\centering
\begin{tabular}{rlrrrrrr}
  \hline
 & method & RMSE & bias & coverage & RMSE & bias & coverage \\
  \hline
1 & naive & 0.38 & 0.10 & 0.95 & 0.42 & 0.21 & 0.90 \\
  2 & Bayes & 0.29 & -0.06 & 0.95 & 0.29 & -0.01 & 0.95 \\
   \hline
\end{tabular}
\end{table}
```

4.5.2.2 Average effect in the population In Table 2 we show the mean squared error (MSE) and coverage for estimating μ_i, which is the average effect in similar trials. We see much better performance of the Bayesian approach compared to naively using the unbiased estimator and it confidence interval. We do note that the coverage of the Bayesian credible interval is clearly short of nominal. This is likely due to the reasons mentioned above.

```r
par=sim$mu
b=sim$b
se=sim$s
est=sim$muhat
L=sim$muhat_L
U=sim$muhat_U

tab1=maketable(par,b,se,est,L,U)

par=sig$mu
b=sig$b
se=sig$s
est=sig$muhat
L=sig$muhat_L
U=sig$muhat_U

tab2=maketable(par,b,se,est,L,U)


caption="The mean squared error and coverage for estimating $\\mu_i$, i.e. the
average effect in similar trials. On the right-hand side of the table, we condition
on statistical significance, i.e. $|b_i/s_i| > 1.96$."

tab=cbind(tab1,tab2[2:4])
kable(tab,caption=caption,label="tab:mu") %>%
  add_header_above(header=c(" "=1,"all"=3,"significant only"=3)) %>%
  kable_styling(latex_options = "HOLD_position")
```

Table 3: The mean squared error and coverage for estimating μ_i, i.e. the average effect in similar trials. On the right-hand side of the table, we condition on statistical significance, i.e. $|b_i/s_i| > 1.96$.

	all			significant only		
method	RMSE	bias	coverage	RMSE	bias	coverage
naive	0.53	0.15	0.82	0.72	0.41	0.63
Bayes	0.35	-0.11	0.93	0.38	-0.03	0.94

```r
xtable(tab)
```

```
% latex table generated in R 4.2.2 by xtable 1.8-4 package
% Mon Dec  2 11:37:39 2024
\begin{table}[ht]
\centering
\begin{tabular}{rlrrrrrr}
  \hline
 & method & RMSE & bias & coverage & RMSE & bias & coverage \\
  \hline
1 & naive & 0.53 & 0.15 & 0.82 & 0.72 & 0.41 & 0.63 \\
  2 & Bayes & 0.35 & -0.11 & 0.93 & 0.38 & -0.03 & 0.94 \\
   \hline
\end{tabular}
```

\end{table}

In particular, the difference in MSE is

```r
mean((sim$mu - sim$b)^2 - (sim$mu - sim$muhat)^2)
```

```
## [1] 0.1627914
```

5 Frequentist perspective

5.1 Bias

```r
sim$bias1=sim$betahat - sim$beta
sim$bias2=sim$muhat - sim$mu

# sample_n(.x, 10000) is to reduce size of PDF graphs

p1=ggplot(sim,aes(x=beta,y=bias1)) +
  geom_point(data = ~ sample_n(.x, 10000), size=0.5,alpha=0.5,color="grey") +
  geom_abline(intercept = 0, slope = 0, linetype="dashed") +
  geom_smooth(method="loess",formula="y ~ x",linewidth=0.5,color="black") +
  xlim(-3,3) + ylim(-3,3) + xlab("beta*") + ylab("error for beta*") +
  theme_bw()

p2=ggplot(sim, aes(x = mu,y=bias2)) +
  geom_point(data = ~ sample_n(.x, 10000), size=0.5,alpha=0.5,color="grey") +
  geom_abline(intercept = 0, slope = 0, linetype="dashed") +
  geom_smooth(method="loess",formula="y ~ x",linewidth=0.5,color="black") +
  xlim(-3,3) + ylim(-3,3) + xlab("mu*") + ylab("error for mu*") +
  theme_bw()

p3=ggplot(sim,aes(x=b,y=bias1)) +
  geom_point(data = ~ sample_n(.x, 10000), size=0.5,alpha=0.5,color="grey") +
  geom_abline(intercept = 0, slope = 0, linetype="dashed") +
  geom_smooth(method="loess",formula="y ~ x",linewidth=0.5,color="black") +
  xlim(-3,3) + ylim(-3,3) + xlab("b*") + ylab("") +
  theme_bw()

p4=ggplot(sim, aes(x = b,y=bias2)) +
  geom_point(data = ~ sample_n(.x, 10000), size=0.5,alpha=0.5,color="grey") +
  geom_abline(intercept = 0, slope = 0, linetype="dashed") +
  geom_smooth(method="loess",formula="y ~ x",linewidth=0.5,color="black") +
  xlim(-3,3) + ylim(-3,3) + xlab("b*") + ylab("") +
  theme_bw()

plot_grid(p1, p3, p2, p4, nrow=2,axis="lr",align="v")
```

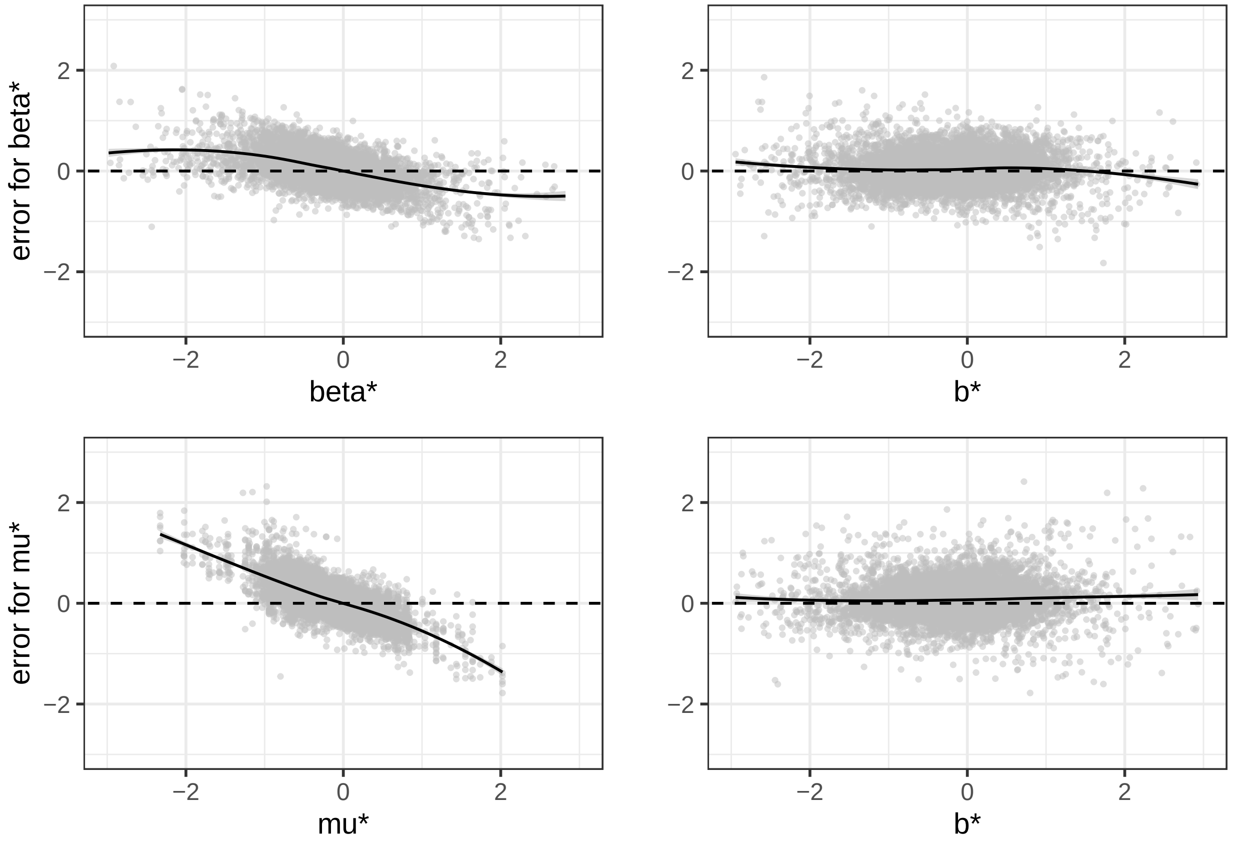


```
ggsave("figures/bias.pdf",width = 8, height = 5)
ggsave("figures/bias.png",width = 8, height = 5,dpi=300)
```

5.2 Difference in MSE

```
sim$diffSE1=(sim$beta - sim$b)^2 - (sim$beta - sim$betahat)^2
sim$diffSE2=(sim$beta - sim$b)^2 - (sim$beta - sim$betahat)^2

p1=ggplot(sim,aes(x=beta,y=diffSE1)) +
  geom_point(data = ~ sample_n(.x, 10000), size=0.5,alpha=0.5,color="grey") +
  geom_abline(intercept = 0, slope = 0, linetype="dashed") +
  geom_smooth(method="loess",formula="y ~ x",linewidth=0.5,color="black") +
  xlim(-3,3) + ylim(-3,3) + xlab("beta*") + ylab("estimating beta*") +
  theme_bw()

p2=ggplot(sim, aes(x = mu,y=diffSE2)) +
  geom_point(data = ~ sample_n(.x, 10000), size=0.5,alpha=0.5,color="grey") +
  geom_abline(intercept = 0, slope = 0, linetype="dashed") +
  geom_smooth(method="loess",formula="y ~ x",linewidth=0.5,color="black") +
  xlim(-3,3) + ylim(-3,3) + xlab("mu*") + ylab("estimating mu*") +
  theme_bw()


p3=ggplot(sim,aes(x=b,y=diffSE1)) +
```

```
  geom_point(data = ~ sample_n(.x, 10000), size=0.5,alpha=0.5,color="grey") +
  geom_abline(intercept = 0, slope = 0, linetype="dashed") +
  geom_smooth(method="loess",formula="y ~ x",linewidth=0.5,color="black") +
  xlim(-3,3) + ylim(-3,3) + xlab("b*") + ylab("") +
  theme_bw()

p4=ggplot(sim, aes(x = b,y=diffSE2)) +
  geom_point(data = ~ sample_n(.x, 10000), size=0.5,alpha=0.5,color="grey") +
  geom_abline(intercept = 0, slope = 0, linetype="dashed") +
  geom_smooth(method="loess",formula="y ~ x",linewidth=0.5,color="black") +
  xlim(-3,3) + ylim(-3,3) + xlab("b*") + ylab("") +
  theme_bw()

p = plot_grid(p1, p3, p2, p4, nrow=2,axis="lr",align="v")
title = ggdraw() + draw_label("difference in squared errors", fontface='bold')
plot_grid(title,p,ncol=1, rel_heights=c(0.1, 1))
```

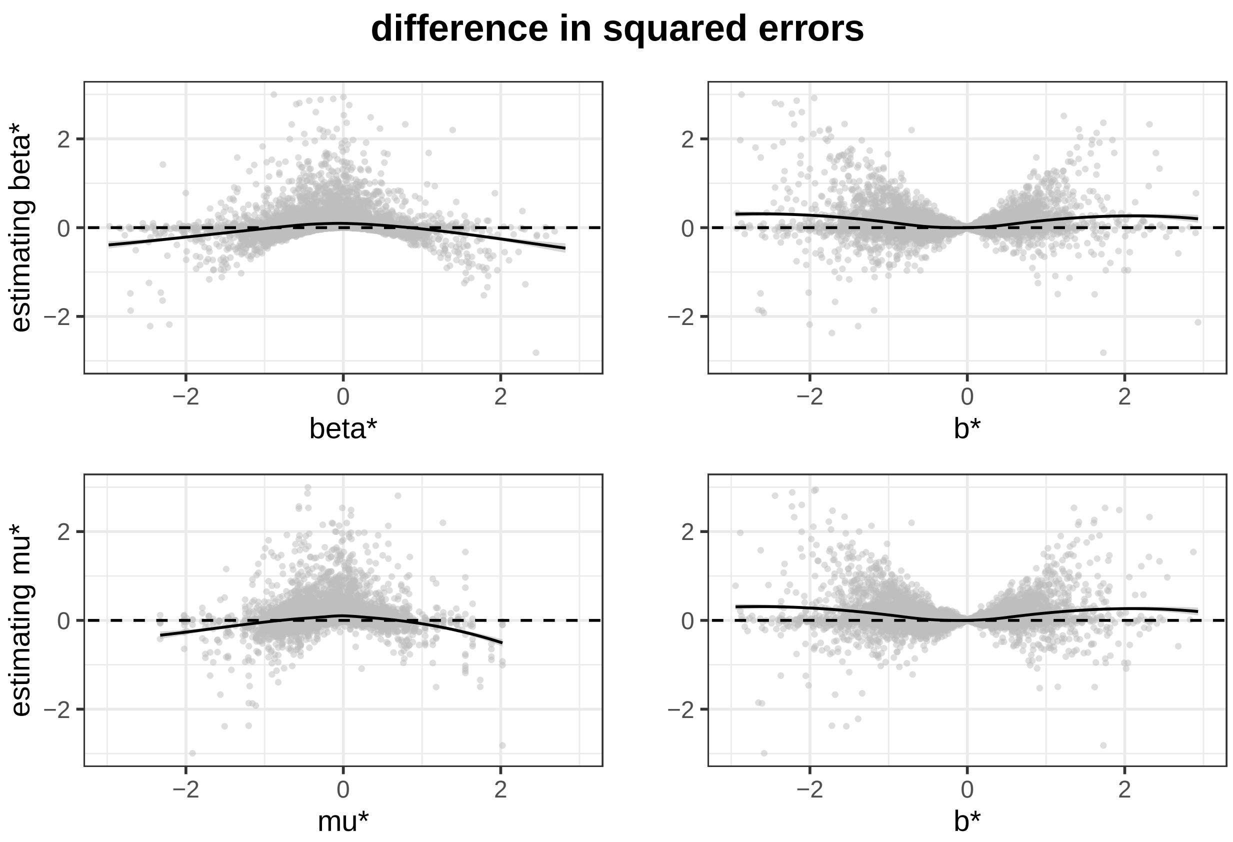


```
ggsave("figures/diff_error2.pdf",width = 8, height = 5)
ggsave("figures/diff_error2.png",width = 8, height = 5,dpi=300)
```
